# Relationship between preoperative high arterial blood lactate level and delirium after deep brain stimulation surgery in Parkinson’s disease

**DOI:** 10.3389/fragi.2025.1538012

**Published:** 2025-03-25

**Authors:** Wenbin Lu, Miaomiao Rao, Liangliang Lu, Panpan Li, Xiaorong Dou, Jinjun Bian, Xiaoming Deng

**Affiliations:** Faculty of Anesthesiology, Changhai Hospital, Naval Military Medical University, Shanghai, China

**Keywords:** Parkinson’s disease, subthalamic nucleus, deep brain stimulation, postoperative delirium, lactate level

## Abstract

**Introduction:** We performed the retrospective study to investigate the relationship between preoperative arterial blood lactate level and postoperative delirium (POD) in Parkinson's disease (PD) patients undergoing deep brain stimulation (DBS) surgery.

**Methods:** Perioperative data of patients undergoing DBS surgery under total intravenous anesthesia were collected in the study. In addition, mini-mental state exam score for assessing cognitive function and confusion assessment method for assessing perioperative delirium in the PD patients were collected. The relationship between preoperative lactate level and POD was analyzed using binary logistic regression analysis.

**Results:** A total of 156 patients were included, of whom 29 (17.6%) patients developed POD. Multivariable logistic regression analysis showed that preoperative lactate level was independently associated with POD regarding of continuous variable [odds ratio (OR) = 12.46, 95% confidence interval (CI)=3.12–49.71, P<0.001] or categorical variable (OR= 3.58, 95% CI =1.20–10.65, P=0.022 for lactate≥1.41). Receiver operating characteristic curve analysis showed that preoperative arterial blood lactate level was a significant predictive biomarker for POD, with an area under the curve of 0.708(95%CI=0.606–0.809, P<0.05). Subgroup analysis indicated that high preoperative lactate levels were an independent risk factor for delirium after DBS surgery (OR=10.71,95%CI=1.17–97.87, P=0.036) in female Parkinson's disease patients.

**Discussion:** Preoperative high level of lactate is an independent factor for delirium after DBS surgery in patients with Parkinson's disease.

## 1 Introduction

Parkinson’s disease (PD) is a progressive neurodegenerative disorder characterized by motor and non-motor symptoms, with advanced stages often necessitating deep brain stimulation (DBS) surgery for symptom management (Jost et al., 2024; Zampogna et al., 2024). While DBS is an effective intervention for motor complications in PD, the occurrence of postoperative delirium (POD) poses a significant challenge in the management of these patients (Lu et al., 2022; Lu et al., 2024; Zhou et al., 2023). POD is associated with prolonged hospitalizations, cognitive decline, and increased healthcare costs (Gjini et al., 2024; Kunicki et al., 2023). Numerous animal and clinical studies have explored the pathogenesis of POD (Lu et al., 2022; Zhang et al., 2021), yet effective methods for preventing and treating POD are currently lacking. Therefore, early identification of the risk factors for delirium after DBS surgery in PD patients can play a vital role in the perioperative management of POD. Increasing studies have shown age, cognitive function, and PD-related scores are associated with delirium after DBS surgery (Astalosch et al., 2024; Zhou et al., 2022).

Previous studies have shown that lactate, as a product of anaerobic metabolism, not only reflects tissue hypoperfusion (Wardi et al., 2020) but is also associated with inflammation in the body (Chen et al., 2024; Huang et al., 2024). A retrospective investigation showed that higher lactate level was significantly associated with multiple organ failure in severely injured blunt trauma patients. (Richards et al., 2018). In addition, a study from Pediatric Intensive Care database found that a non-linear correlation between lactate levels and prognosis in pediatric sepsis, where elevated lactate was associated with higher 28-day mortality (Song et al., 2024). Moreover, community-based cross-sectional study with 2,523 Chinese adults indicated that increased lactate was associated with mild cognitive impairment (Pan et al., 2019). However, the specific relationship between preoperative arterial blood lactate levels and the incidence of delirium in PD patients undergoing DBS surgery remains unclear.

In this study, we hypothesized that elevated preoperative arterial blood lactate levels would be independently associated with increased risk of delirium after DBS surgery in PD patients. To investigate this link, we systematically evaluated the association between preoperative lactate levels and POD. Our findings may provide valuable insights into the pathophysiology of delirium in PD patients undergoing DBS surgery and inform strategies for risk stratification and preventive interventions in this vulnerable population.

## 2 Material and methods

### 2.1 Study design and participants

Our retrospective study included PD patients who underwent elective subthalamic nucleus-DBS surgery under total intravenous anesthesia between January 2021 and January 2023 at the Department of Anesthesiology of Changhai Hospital. The protocol of the study was approved by the ethics committee of our hospital with approval number: CHEC 2020-151. Informed consent was waived by the ethics committee due to the retrospective study.

Participants undergoing DBS surgery for the first time were included in the study. Inclusion criteria including: age ≥55 and American Society of Anesthesiologists (ASA) classification I-III. Patients with delirium, liver function abnormalities, and missing lactate value before surgery were excluded.

### 2.2 Anesthesia and surgery

Surgical candidacy for DBS was determined using education-stratified Mini-Mental State Examination (MMSE) thresholds validated in the Chinese population. The cut-off of the MMSE score are as follows: MMSE ≥16 for illiterate individuals, MMSE ≥19 for those with 1–6 years of education, and MMSE ≥23 for those with >6 years of education (Liu et al., 2015). The patients underwent two-stage DBS implantation under general anesthesia, which was consistent with the previous study (Lei et al., 2022). Head frame was installed under local anesthesia in the ward, the STN targets were located under MRI, and their three-dimensional coordinates were calculated.

In the operating room, electrode and pulse generator implantation were performed in general anesthesia. Patients received standard intraoperative monitoring (non-invasive blood pressure, heart rate, pulse oximetry saturation, and end-tidal carbon dioxide partial pressure) and anesthesia induction with dexamethasone (8 mg), sufentanil (0.3–0.5 μg kg^-1^), propofol (2–3 mg kg^-1^), and rocuronium (0.6 mg kg^-1^). Anesthesia was sustained with propofol (6–8 mg kg^-1^ h^-1^) and remifentanil (0.1–0.2 μg kg^-1^ min^-1^) to stabilize hemodynamics. Thirty minutes before surgery completion, sufentanil (10 µg) was administered. Patients with postoperative numerical rating scale (NRS) pain score ≥4 were administered parecoxib sodium 40 mg as the rescue treatment. Patients experienced nausea or vomiting were administered ondansetron 4 mg.

### 2.3 Data collection

Demographic and clinical data were collected from medical records, including age, gender, body mass index, ASA classification, levodopa equivalent dose, preoperative MMSE score, level of education, surgery duration; preoperative comorbidities (coronary heart disease, hypertension, diabetes mellitus), and preoperative laboratory tests, including blood leukocyte count, lymphocyte count, neutrophil count, albumin level, D-dimer level, arterial blood lactate level. The arterial blood lactate levels were measured using the enzymatic colorimetric assay.

Additionally, PD-related symptoms were also collected, including Non-Motor Symptom Scale (NMSS) score, Hamilton Depression Scale (HAMD) score, KINGS Parkinson’s Disease Pain Scale (KPPS) score, Hamilton Anxiety Scale (HAMA) score, and Movement Disorder Society-Unified Parkinson’s Disease Rating Scale (MDS-UPDRS) score. The raters for collecting the PD-related symptoms score were blinded to the group classification. We further documented perioperative variables, including intraoperative hypotension (defined as mean arterial pressure less than 65 mmHg for >1 min), total morphine milligram equivalent, propofol consumption, postoperative 24-h NRS pain scores, rescue analgesic requirements, and ondansetron administration within the 24-h after surgery. The missing values for variables with less than 5% missing data were handled using multiple imputation.

Delirium was assessed preoperatively and for 3 days postoperatively (before 10 a.m. and after 5 p.m. for each day) using the Confusion Assessment Method (CAM), which is a commonly used method for diagnosing delirium with a sensitivity of 94% and a specificity of 89% (Yuan et al., 2020). Delirium was diagnosed when a patient presented with acute onset, fluctuating course, and poor concentration, along with confused thinking or altered state of consciousness (Mi et al., 2024). Cognitive screening was performed using MMSE scale at admission. The assessment of CAM questionnaire and MMSE scale is performed by doctors who have received training and are qualified in CAM and MMSE.

### 2.4 Statistical analysis

Descriptive statistics were performed for the two groups based the delirium after surgery. Continuous data with a normal distribution are presented as mean ± standard deviation, analyzed using independent sample t-tests. Data with non-normal distribution are presented as median (interquartile range), analyzed using the Mann-Whitney U test. Categorical data are presented as frequencies (percentages), analyzed using the chi-square test or Fisher’s exact test.

Binary logistic regression analysis was conducted to explore the relationship between preoperative lactate levels and POD. Lactate levels were categorized as low lactate levels (<1.41 mmol/L) and high lactate levels (≥1.41 mmol/L) based on the median. Variables with *P* < 0.05 in univariate analysis were included in the multivariable logistic regression analysis. The association between lactate levels and POD were evaluated when lactate levels were both continuous and binary variables. Model I did not adjust for any variables; Model II adjusted for variables with *P* < 0.05 and change in odds ratio of at least 10% after inclusion; Model III adjusted for all variables with *P* < 0.05 in univariate analysis. In addition, receiver operating characteristic curve analysis was conducted to assess the predictive power of lactate for delirium after DBS surgery.

Subgroup analysis was performed based on age (<65 years or ≥65 years), gender, and preoperative MMSE score (<27 or ≥27) to evaluate the relationship between lactate levels (as a binary variable) and POD. Subgroup analysis adjusted for all variables with *P* < 0.05 in univariate analysis, excluding stratification factor itself. The Free Statistics software version 1.7.1 and the software package R were used to conduct all statistical analyses. A *P*-value of <0.05 was considered statistically significant.

## 3 Results

**FIGURE 1:**
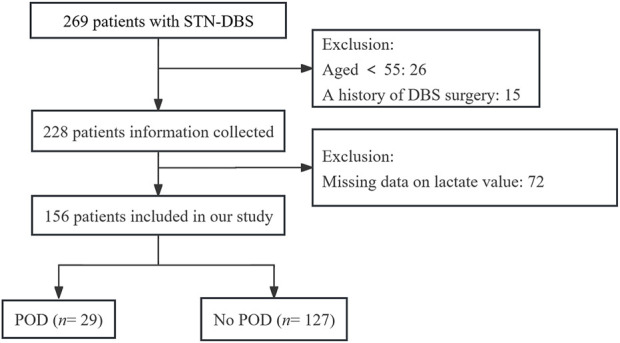
Flowchart of study population.

A total of 228 PD patients undergoing elective DBS surgery met the inclusion criteria, of which 72 patients were excluded due to missing arterial blood lactate values. Ultimately, statistical analysis was conducted on 156 PD patients, including 78 females and 78 males. Among the 156 PD patients, 29 patients (17.6%) experienced POD. Figure 1 shows the participant flow diagram.

### 3.1 Participants characteristics

Table 1 indicates that patients with POD were older, had a higher prevalence of diabetes, lower preoperative MMSE scores (all *P* < 0.05) than patients without POD. Additionally, compared with patients without POD, patients with POD had a worse PD-related scores, including higher NMSS scores, higher UPDRS part I, part II, and part III scores (all *P* < 0.05). Importantly, patients with POD had a higher blood lactate levels (*P* < 0.001) compared to patients without POD. No statistical differences were observed in the comparison of other baseline characteristics between the two groups.

**TABLE 1:** Baseline clinical characteristics of the study patients.

	Non-POD group (n = 127)	POD group (n = 29)	*P*
Age (years)	62.54 ± 6.67	68.24 ± 6.67	<0.001
Female	68 (53.54)	10 (34.48)	0.064
BMI (kg/m^2^)	22.83 ± 3.34	23.86 ± 3.52	0.141
ASA physical status			0.093
II	98 (77.17)	18 (62.07)	
III	29 (22.83)	11 (37.93)	
Education level			0.339
Illiterate school	13 (10.24)	3 (10.34)	
Primary school	22 (17.32)	9 (31.03)	
Middle school	62 (48.82)	13 (44.83)	
Technical secondary school or more	30 (23.62)	4 (13.79)	
Levodopa equivalent dose (mg/d)	750.00 (577.10, 900.00)	750.00 (633.30, 750.00)	0.537
Hypertension	34 (26.77)	10 (34.48)	0.405
Diabetes	7 (5.51)	5 (17.24)	0.048
Coronary heart disease	1 (0.79)	1 (3.45)	0.338
Operation time (min)	135.00 (125.00, 145.00)	140.00 (130.00, 155.00)	0.05
Preoperative MMSE score	27.00 (25.00, 28.00)	23.00 (19.00, 27.00)	<0.001
NMSS score	17.00 (15.00, 20.00)	19.00 (17.00, 20.00)	0.016
KPPS score	8.00 (4.00, 16.00)	9.00 (6.00, 14.00)	0.477
HADM score	11.00 (8.00, 15.00)	12.00 (10.00, 17.00)	0.143
HAMA score	9.00 (6.00, 12.50)	10.00 (7.00, 13.00)	0.185
UPDRS part 1 score	18.00 (14.00, 20.50)	21.00 (19.00, 23.00)	0.001
UPDRS part 2 score	25.00 (20.50, 28.00)	29.00 (25.00, 37.00)	0.001
UPDRS part 3 (off state) score	59.00 (51.00, 70.00)	66.00 (56.00, 81.00)	0.016
UPDRS part 3 (on state) score	25.00 (18.00, 35.00)	31.00 (25.00, 49.00)	0.006
UPDRS part 4 score	8.00 (7.00, 10.00)	9.00 (7.00, 10.00)	0.561
Intraoperative hypotension	28 (22.0)	8 (27.6)	0.523
Morphine milligram equivalent (mg)	185.00 (160.00, 205.00)	190.00 (175.00, 200.00)	0.529
Propofol (mg)	750.00 (620.00, 855.00)	780.00 (650.00, 900.00)	0.470
Postoperative 24-h NRS score	1.00 (1.00, 2.00)	1.00 (1.00, 3.00)	0.408
Rescue analgesic administration	22 (17.30)	8 (27.60)	0.206
Ondansetron administration	26 (36.10)	9 (47.40)	0.370
Lactate (mmol/L)	1.40 (1.08, 1.70)	1.70 (1.40, 2.00)	<0.001
Leukocyte count (10^9^/L)	5.38 (4.57, 6.41)	5.07 (4.44, 6.60)	0.654
Lymphocyte count (10^9^/L)	1.62 (1.29, 2.00)	1.32 (1.12, 1.83)	0.085
Neutrophil count (10^9^/L)	3.21 (2.66, 3.83)	3.23 (2.61, 4.72)	0.604
Albumin (g/L)	44.00 (42.00, 46.00)	43.00 (41.00, 45.00)	0.170
D-dimer (mg/L)	0.35 (0.28, 0.46)	0.45 (0.31, 0.55)	0.127

Data are reported as mean ± standard deviation, frequency (%), or median (inter-quartile range). POD, postoperative delirium; BMI, body mass index; ASA, american society of anesthesiologists; MMSE, Mini-mental State Examination; NMSS, non-motor symptom scale; KPPS, KINGS, Parkinson’s disease pain scale; HADM, hamilton depression scale; HAMA, hamilton anxiety scale; UPDRS, unified Parkinson’s disease rating scale; NRS, numeric rating scale.

### 3.2 Relationship between lactate and POD

**FIGURE 2:**
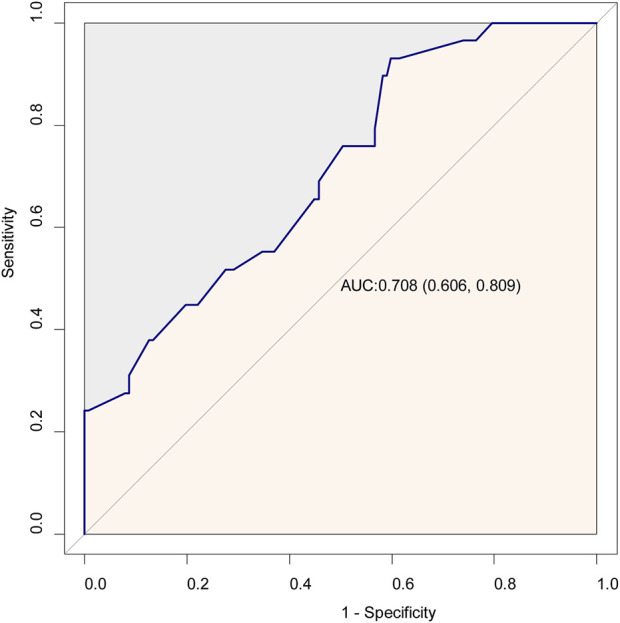
Receiver operating characteristic curve for lactate as a predictor of POD.

Variables with *P* < 0.05 in Table 1 were included in the multivariable logistic regression analysis. In Model I without adjusting for any variables, the logistic regression analysis indicated that preoperative lactate level, as a continuous variable, was positively correlated with POD (OR = 7.10, 95% CI = 2.60–19.39, *P* < 0.001); when considered as a binary variable, high lactate level was also associated with POD (OR = 2.64, 95% CI = 1.12–6.25, *P* = 0.027). In addition, in Model III after adjusting for all variables with P < 0.05, preoperative lactate level remained independently associated with POD, whether as a continuous variable (OR = 12.46, 95% CI = 3.12–49.71, *P* < 0.001) or a binary variable (OR = 3.58, 95% CI = 1.20–10.65, *P* = 0.022) (Table 2). Sensitivity analysis using receiver operator characteristics curve (ROC) defined lactate threshold (1.15 mmol/L) further validated the stability of this relationship (Supplementary Table S1). Moreover, ROC analysis revealed that the lactate level was a moderate but statistically significant predictive biomarker for POD, with an area under the curve of 0.708 (95% CI = 0.606–0.809, *P* < 0.05) (Figure 2).

**TABLE 2:** Univariable and multivariable logistic regression analysis to assess the association between lactate and delirium after DBS surgery.

Variable	Model I		Model II		Model III	
	OR (95% CI)	*P*	OR (95% CI)	*P*	OR (95% CI)	*P*
Lactate	7.10 (2.60–19.39)	<0.001	10.61 (3.14–35.86)	<0.001	12.46 (3.12–49.71)	<0.001
Lactate <1.41	Ref		Ref		Ref	
Lactate≥1.41	2.64 (1.12–6.25)	0.027	3.04 (1.13–8.19)	0.027	3.58 (1.2–10.65)	0.022

Model I, adjusted for nothing; model II, adjusted for age, preoperative MMSE, score; model III, adjusted for model II, diabetes; NMSS, score, and UPDRS, part 1, 2, and 3 scores; OR, odd ratio; CI, confidence interval; Rf, reference; MMSE, Mini-mental State Examination; NMSS, non-motor symptom scale; and UPDRS, unified Parkinson’s disease rating scale.

### 3.3 Subgroup analysis

Subgroup analysis revealed no interaction between preoperative lactate levels and the subgroups. However, high preoperative lactate levels were identified as an independent risk factor for delirium after DBS surgery (OR = 10.71, 95% CI = 1.17–97.87, *P* = 0.036) in female Parkinson’s disease patients (Table 3).

**TABLE 3:** Association between lactate and delirium after DBS surgery in subgroups.

Subgroup	Patients	POD (%)	Lactate		*P* for interaction
			<1.41	≥1.41	
Age					0.747
Age <65	85	10 (11.76)	Ref	2.73 (0.49–15.23)	
Age ≥65	71	19 (26.76)	Ref	3.78 (0.85–16.81)	
Gender					0.265
Female	78	10 (12.82)	Ref	10.71 (1.17–97.87)	
Male	78	19 (24.36)	Ref	2.47 (0.56–10.88)	
Preoperative MMSE score					0.694
MMSE<27	73	21 (28.76)	Ref	4.19 (0.92–19.07)	
MMSE≥27	83	8 (9.64)	Ref	2.44 (0.31–19.48)	

ORs (95% CIs) were derived from logistic regression models. Covariates were adjusted by age, preoperative MMSE, score, diabetes; NMSS, score, and UPDRS, part 1, 2, and 3 scores in each subgroup, excluding stratification factor itself. POD, postoperative delirium; MMSE, Mini-mental State Examination; NMSS, non-motor symptom scale; and UPDRS, unified Parkinson’s disease rating scale; Rf, reference.

## 4 Discussion

This study investigated for the first time the relationship between preoperative lactate levels and POD in Parkinson’s disease patients undergoing DBS surgery. We found that the incidence of POD in Parkinson’s disease patients was 17.6%. Patients who experienced POD were older, had a higher prevalence of diabetes, lower preoperative MMSE scores, higher NMSS scores, higher UPDRS part I, II, III scores, and higher blood lactate levels. Importantly, our study revealed that high preoperative lactate level was an independent risk factor for POD in Parkinson’s disease patients undergoing DBS surgery, whether considered as a continuous variable or a binary variable. Subgroup analysis also indicated that in female Parkinson’s disease patients, high preoperative lactate level was an independent risk factor for delirium after DBS surgery.

Blood lactate, as a marker of metabolism and inflammation, can not only predict outcomes in critically ill patients but is also associated with neurobehavioral symptoms. Study has shown that elevated blood lactate level increased the short-term and long-term mortality risk in patients with acute pancreatitis (Zeng et al., 2024). And higher blood lactate level was associated with POD in elderly trauma patients, especially hip fracture (Lee et al., 2019; X. H; Liu et al., 2023). Additionally, as a marker of mitochondrial dysfunction, elevated blood lactate level was related to depressive symptoms in patients with bipolar disorder (Jeong et al., 2020).

Previous study also showed that high blood lactate level was associated with mild cognitive impairment in community-dwelling older adults (Pan et al., 2019). Consistent with previous research, our study found that patients with POD had higher preoperative blood lactate level compared to patients without POD. Moreover, after adjusting for other confounding factors, high lactate level was independently associated with POD in Parkinson’s disease patients undergoing DBS surgery. However, ROC analysis indicated that lactate level alone may not be sufficient as a predictive biomarker, with an area under the curve of 0.708.

In addition, recent studies have shown that inflammatory markers and neurophysiological markers were associated with delirium after surgery (Cao et al., 2024; Hight et al., 2024), which partially aligns with our study, as all indicating systemic or cerebral stress contributes to delirium after surgery. However, high lactate level may more directly reflect preoperative metabolic disturbances in PD patients. Our study expands the mechanistic understanding of delirium after surgery by emphasizing metabolic dysregulation as a complementary pathway to inflammation or neurophysiological dysfunction.

However, the specific reasons and mechanisms underlying the association between high lactate level and delirium after DBS surgery remain unclear. Current animal and clinical studies suggest that surgery-anesthesia induced inflammatory responses play a vital role in the development of POD (Noah et al., 2021; Zhang et al., 2022). Lactate is produced from pyruvate via the lactate dehydrogenase in the glycolytic pathway, which increases inflammatory cytokine production. In addition, increased lactate indicates mitochondrial dysfunction during resting conditions (Esmael et al., 2021). Therefore, blood lactate can serve as a marker of mitochondrial dysfunction and inflammatory response. Which is involved in the pathogenesis of various inflammatory diseases, including delirium after surgery (Dai et al., 2023; Li et al., 2023; Yang et al., 2024).

Importantly, previous study showed that mitochondrial dysfunction promoted central nervous system inflammation, leading to cognitive impairment (Cossu and Hattori, 2024). As a potential pro-inflammatory signaling molecule, lactate increases mitochondrial reactive oxygen species production and promotes the production of pro-inflammatory cytokines in the body (Pucino et al., 2018). Inflammatory reactions play a crucial role not only in the pathogenesis of cognitive impairment but also in the development of POD (Zhang, et al., 2022; Song et al., 2022). Therefore, higher lactate level may contribute to the occurrence and progression of POD by increasing central nervous system inflammation. Besides, the use of lactate as a predictor of POD may have an important role in the clinical settings due to its simplicity to obtain and ease of clinical use, which is not subject to individual subjectivity.

This study has certain limitations. Firstly, our study is a small-sample, single-center retrospective study that only included Parkinson’s patients undergoing DBS surgery under total intravenous anesthesia. Secondly, we only collected arterial blood lactate values preoperatively. Further research is needed to explore the association between POD and the dynamic changes of blood lactate. Thirdly, we only included the patients with late-onset Parkinson’s disease (age ≥55 years), limiting the generalizability of the results. Fourthly, the association between lactate level and delirium after surgery in female subgroup should be interpreted with caution due to limited sample size. Lastly, it may be more sensitive to use a variety of cognitive scoring scales (including Montreal cognitive assessment) than the MMSE score with ceiling effect and poor sensitivity for mild cognitive impairment in the present study. Therefore, a multicenter large-sample observational study is required to validate the relationship between preoperative blood lactate and POD.

## 5 Conclusion

In summary, our study found that higher preoperative blood lactate level is an independent risk factor for POD in Parkinson’s patients undergoing DBS surgery. Therefore, early identification of patients with elevated preoperative blood lactate level may help in the early detection and reduction of POD in Parkinson’s patients undergoing DBS surgery.

## Data Availability

The raw data supporting the conclusions of this article will be made available by the authors, without undue reservation.
