# Supplementary material for: Relationship between preoperative high arterial blood lactate level and delirium after deep brain stimulation surgery in Parkinson’s disease
Source: Front Aging. 2025 Mar 25;6:1538012. doi: 10.3389/fragi.2025.1538012 (PMC11975659; doi:10.3389/fragi.2025.1538012)
Supplement: Supplementary file 1 [file DataSheet1.docx]

Supplementary Material

Relationship between preoperative high arterial blood lactate level and delirium after deep brain stimulation surgery in Parkinson’s disease

Wenbin Lu ^1,^ ^*^, Miaomiao Rao ^1, *^, Liangliang Lu ^1^, Panpan Li ^1^, Xiaorong Dou ^1^, Jinjun Bian ^1,^ ^#^ Xiaoming Deng ^1, #^

^1^ Faculty of Anesthesiology, Changhai Hospital, Naval Medical University/Second Military Medical University, PLA, Shanghai, 200433, China

^*^ These authors contributed equally to this work

^#^ Corresponding author

Correspondence:

Jinjun Bian, M.D., Ph.D.

Faculty of Anesthesiology, Changhai Hospital, Naval Medical University,

168, Changhai Road, 200433, Shanghai, China.

Tel: +86-021-31161841

Fax: +86-021-31161841

E-mail: jinjunbicu@163.com

Xiaoming Deng, Ph.D.

Faculty of Anesthesiology, Changhai Hospital, Naval Medical University,

168, Changhai Road, 200433, Shanghai, China.

Tel: +86-021-31161837

Fax: +86-021-31161837

E-mail: dengphd@smmu.edu.cn

Supplementary Table 1 Univariable and multivariable logistic regression analysis to assess the association between high lactate (≥1.15mmol/L based ROC-derived cut-off value) and delirium after DBS surgery.

| Variable | Model I | |  | Model II | |  | Model III | |
| --- | --- | --- | --- | --- | --- | --- | --- | --- |
|  | OR (95% CI) | *P* |  | OR (95% CI) | *P* |  | OR (95% CI) | *P* |
| Lactate ＜1.15 | Ref |  |  | Ref |  |  | Ref |  |
| Lactate≥1.15 | 9.06 (2.06~39.76) | 0.003 |  | 15.47 (2.67~89.72) | 0.002 |  | 12.34 (2.26~67.36) | 0.004 |

Model I, adjusted for nothing; model II, adjusted for age, preoperative MMSE score; model III, adjusted for model II, diabetes, NMSS score, and UPDRS part 1, 2, and 3 scores. OR, odd ratio; CI, confidence interval; Rf, reference; MMSE, Mini-mental State Examination; NMSS, non-motor symptom scale; and UPDRS, unified Parkinson’s disease rating scale.
